# Interference with ANXA8 inhibits the malignant progression of ovarian cancer by suppressing the activation of the Wnt/β-catenin signaling pathway via UCHL5

**DOI:** 10.18632/aging.205991

**Published:** 2024-07-26

**Authors:** Li Xu, Liang Wang, Yaping Gan, Jiazhi Lin, Shuting Ning, Jinjin Deng, Yingxia Ning, Weifeng Feng

**Affiliations:** 1 Department of Gynaecology and Obstetrics, The First Affiliated Hospital of Jinan University, Guangzhou 510632, Guangdong, China; 2 Department of Gynaecology and Obstetrics, The First Affiliated Hospital of Guangzhou Medical University, Guangzhou 510120, Guangdong, China; 3 Department of Traditional Chinese Medicine, The First Affiliated Hospital of Jinan University, Guangzhou 510632, Guangdong, China; 4 Guangdong Guojian Pharmaceutical Consulting Co., Ltd., Guangzhou 510030, China

**Keywords:** ovarian cancer, ANXA8, proliferation, invasion, migration

## Abstract

Ovarian cancer (OC), which threatens women’s lives, is a common tumor of the female reproductive system. Annexin A8 (ANXA8) is highly expressed in OC. However, the mechanism of ANXA8 in OC remains unclear. This study investigated the potential mechanisms of ANXA8 in OC. The expression of ANXA8 in OC cells was determined by qRT-PCR and western blotting. ANXA8 interference plasmid was constructed. Moreover, CCK-8, EDU staining, TUNEL staining, western blotting, wound healing, and transwell assays were used to detect cell proliferation, apoptosis, migration, and invasion, respectively. Next, the relationship between ANXA8 and ubiquitin C-terminal hydrolase L5 (UCHL5) was verified through Co-IP. Finally, western blotting was used to detect the expression of Wnt/β-catenin signaling-related proteins. Additionally, we further interfered ANXA8 in nude mice with OC, and detected the expression of ANXA8, UCHL5 and the signaling pathway-related proteins by immunohistochemistry and western blotting. Our results suggested that ANXA8 expression was significantly increased in OC cells. ANXA8 interference significantly attenuated the proliferative, invasive, and migratory capabilities and promoted the apoptotic ability of OC cells. Moreover, the expression of UCHL5 in OC was significantly increased. ANXA8 bound to UCHL5 in OC cells. Knockdown of ANXA8 attenuated OC cell malignant progression by downregulating the expression of UCHL5. Furthermore, ANXA8 affected the expression of Wnt/β-catenin signaling pathway-related proteins in OC cells via UCHL5. Collectively, ANXA8 interference suppressed the activation of Wnt/β-catenin signaling pathway via UCHL5 to inhibit cell proliferation, invasion, migration and induce cell apoptosis in OC, thus presenting a potential therapeutic strategy for OC treatment.

## INTRODUCTION

Ovarian cancer (OC) represents a common neoplasm of the female reproductive system, ranking fifth among the leading causes of cancer death in women, and posing a serious threat to the lives of women [1, 2]. The majority of patients are typically diagnosed during intermediate and even advanced stages of the disease due to the scarcity of reliable diagnostic indicators in the early stage [3]. Due to the recurrence and drug resistance of OC, most patients will relapse within 5 years after the initial treatment, and the rate of five-year survival is less than 50% [4, 5]. Therefore, it is of great significance to explore the key therapeutic targets for the treatment of OC, so as to improve the lifetime and life quality of OC patients.

Annexin A8 (ANXA8) is a member of Annexin which constitutes a family of calcium-dependent phospholipid-binding proteins that are widely distributed in various plants and animals [6]. It encodes an anticoagulant protein and acts as an indirect thrombin-specific complex [7]. It plays an important regulatory role in a variety of cancers. Since ANXA8 protein is an underlying therapeutic target for a variety of cancers, it has received much attention in the medical community in recent years and may be used as a model for the study of human cancer [8–10]. ANXA8 expression has been shown to be increased in OC tissues, and the higher the expression, the worse the prognosis of OC [9]. However, its specific effect on OC is unknown.

Ubiquitin C-terminal hydrolase-L5 (UCHL5), specifically belonging to the subclass that is responsible for the removal of ubiquitin chains from protein substrates, is a member of the deubiquitinating enzyme family [11]. ANXA8 was predicted to bind to UCHL5 using the Biogrid database. Additionally, it has been reported that higher UCHL5 expression in OC tissues indicates worse prognosis, but it is unknown whether ANXA8 can bind to UCHL5 and participate in the process of OC. Hence, this study explored the regulatory roles of ANXA8 and UCHL5 in OC cell proliferation, invasion and migration and the intrinsic mechanisms between the two, thus establishing a theoretical basis for the targeted treatment of OC.

## MATERIALS AND METHODS

### Cell culture

Human ovarian epithelial cells HOSEpiC (MZ-1311, Ningbo Mingzhou Biotechnology Co., LTD, China) and OC cell lines SKOV3 (MZ-0169, Ningbo Mingzhou Biotechnology Co., LTD, China), OVCAR3 (MZ-1071, Ningbo Mingzhou Biotechnology Co., LTD, China), CAOV3 (MZ-2002, Ningbo Mingzhou Biotechnology Co., LTD, China) and Caov-4 (MZ-1650, Ningbo Mingzhou Biotechnology Co., LTD, China) were used in this study. All Cells were cultured in DMEM medium or RPMI-1640 medium supplemented with 10% fetal bovine serum (FBS).

### Quantitative real-time PCR (qRT-PCR)

Total RNA was isolated from the cells utilizing TRIzol reagent (Invitrogen) according to the instructions of the manufacturer. Complementary DNA was synthesized from 1 μg RNA. Then qRT-PCR was conducted using the SYBR Green Kit on the ABI PRISM 7500 Sequence Detection System as per the standard protocol, employing specific primers. GAPDH served as the normalization control. The relative expression levels were computed by employing the DataAssist software (Applied Biosystems, Foster City, CA, USA) by applying the formula 2^−ΔΔCt^ [12].

### Western blotting

Proteins were extracted with RIPA lysis buffer and homogenized. A BCA kit was then used to detect the protein concentration and a quantity of 30 μg of proteins per well was resolved by 10% SDS-polyacrylamide gel electrophoresis, and the separated proteins were subsequently transferred onto a PVDF membrane. Subsequently, the PVDF membrane was incubated with primary antibodies at 4°C overnight and HRP-conjugated donkey anti-rabbit secondary antibody (Dako) for 1 h after being blocked with 5% BSA. Chemiluminescence (GE Healthcare, Chicago, IL, USA) was employed to visualize the proteins, and the band densities were quantified using the ImageJ software (NIH, Bethesda, MD, USA).

### Plasmid construction and cell transfection

Short hairpin RNA (shRNA) against ANXA8, as well as scramble shRNA (sh-NC) and the overexpression plasmid (pcDNA3.1(+)-UCHL5) constructed by inserting the coding sequence (CDS) of porcine UCHL5 gene into the pcDNA3.1 (+) vector were all obtained from Genechem (Shanghai, China). Cells were transfected with these plasmids using Lipofectamine 2000 (Invitrogen) according to the manufacturer’s instructions. The transfection efficiency was detected by qRT-PCR and western blot analysis 48 h after transfection.

### Cell proliferation

Cell proliferation was assessed utilizing CCK-8 assay (MedChemExpress, China). The cells were added to 96-well plates at a density of 1000 cells/well and corresponding transfection was performed. 10 μL of CCK-8 solvent (Sangon Biotech, Shanghai, China) was added to each well and incubated for 2 h. Finally, absorbance at 450 nm was determined using a microplate reader (Bio–Rad, Berkeley, CA, USA).

### 5-ethynyl-2’ -deoxyuridine (EDU) staining assay

EDU staining was employed to assess cell proliferation, following the standard protocol. Cells were added to 6-well plates at a density of 10^5^ cells/well and corresponding transfection was performed. Then cells were incubated with EDU [20 mmol/L) for 2 h. The cells were immobilized using 4% paraformaldehyde for 20 min at room temperature, and the EDU-positive cells were observed.

### Terminal deoxynucleotidyl transferase dUTP nick end labeling (TUNEL) assay

To assess apoptosis in the cerebral cortex, TUNEL assay was conducted employing the *In Situ* Cell Death Detection Kit (Roche Diagnostics, Basel, Switzerland). The cells were immobilized with 4% formaldehyde for 25 min at 4°C, followed by permeabilization with 0.2% TritonX-100 for 5 min. Subsequently, the cells were equilibrated with 100 μL of Equilibration buffer for 10 min at room temperature. 50 μL of TdT reaction mix was added to label the cells at 37°C for 1 h. The reaction was terminated with SSC buffer, and the nuclei were stained with DAPI. The images were captured using a fluorescence microscope.

### Wound healing and transwell assays

For the wound healing assay, OC cells were plated in 6-well plates pretreated with 0.1% gelatin. Upon reaching 70% confluence, the cells were serum-starved overnight. A sterile plastic pipette tip was used to create a scratch wound in the center of the cell monolayer, followed by the removal of debris through PBS washing. Images of the wounds were captured at 0 h and 24 h. For the transwell invasion assay, 5×10^4^ cells suspended in FBS-free medium were seeded onto the upper chamber of the transwell (8 μm pore size, 6.5 mm diameter; Corning) coated with Matrigel (BD Biosciences). Subsequently, the bottom insert was loaded with 500 μl of medium containing 10% FBS. To assess the invasive capacity, non-invasive cells were gently removed by wiping the membrane’s top surface with cotton swabs, while the invasive cells were stained with crystal violet and calculated.

### Coimmunoprecipitation (Co-IP)

The cells were lysed using an IP-lysis buffer. Cellular debris was removed by high-speed centrifugation, and the supernatant from the cell lysates was incubated overnight with specific antibodies at 4°C. Subsequently, pre-cleared protein A/G beads (GE Healthcare, Chicago, IL, USA) were incubated with the supernatant for 3 h. The captured proteins were then analyzed by western blotting.

### Xenograft tumor model

Female BABL/c nude mice (6-week old) were randomly assigned into three groups (Control, Lv-shRNA-NC and Lv-shRNA- ANXA8 groups). The 5×10^6^ CAOV3 cells per group were subcutaneously implanted into the right flank of nude mice. Tumor size was measured weekly using a vernier caliper, and tumor volumes were subsequently calculated. After 21 days, the mice were humanely euthanized, and tumor weight was ascertained. The *in vivo* animal experiments were conducted adhering to the ethical standards and were approved by Guangzhou Medical University.

### Immunohistochemistry (IHC)

The paraformaldehyde-fixed tumor tissues from mice were embedded in paraffin, and 5 μm-thick transverse sections were mounted onto silane-coated slides. The primary antibodies KI67 and cleaved caspase3 were added to the sections in a 100 μl volume and incubated overnight at 4°C after blocking. Subsequently, the sections were reacted with biotinylated rabbit anti-goat IgG for 20 min at 37°C. The stains were developed using 3 mL of diaminobenzidine (DA1010, Beijing Solarbio Science and Technology Co., Ltd., China) for 5-10 min as a chromogen. Finally, the sections were observed under an optical microscope (Olympus Corporation, Tokyo, Japan).

### Statistics

Data were presented as means ± standard deviation (SD) using GraphPad Prism 8 software (GraphPad, La Jolla, CA, USA). Statistical significance was determined by one-way analysis of variance followed by Tukey’s test, with P<0.05 considered significant.

### Availability of data and materials

The analyzed datasets generated during the present study are available from the corresponding author on reasonable request.

### Consent for publication

All the authors agree to be published.

## RESULTS

### ANXA8 expression was increased in OC cell lines

**Figure 1:**
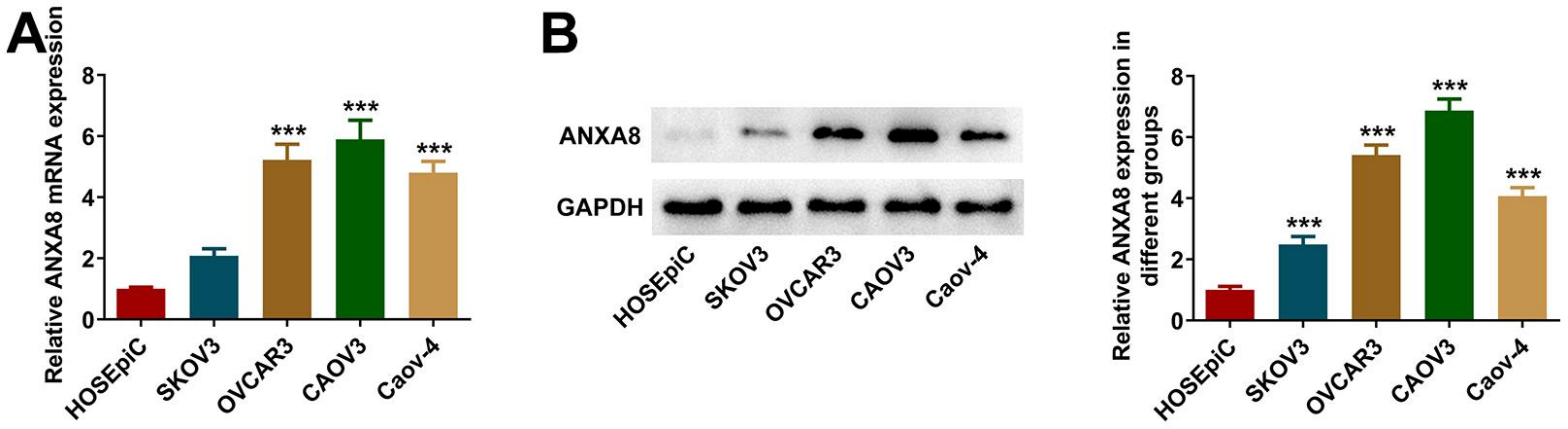
**ANXA8 expression was increased in OC cell lines.** (**A**) The expression of ANXA8 in the OC cell line was detected by RT-qPCR; (**B**) The expression of ANXA8 in the OC cell line was assessed by western blotting. ***P<0.001 vs. HOSEpiC.

The expression of ANXA8 in the OC cell line was detected by RT-qPCR and western blotting. The results showed that the expression of ANXA8 was abnormally elevated in the OC cell lines (Figure 1A, 1B). ANXA8 expression was most significantly elevated in CAOV3 cells, so we selected CAOV3 cells for follow-up experiments.

### Interference with ANXA8 inhibited the proliferation and induced the apoptosis of CAOV3 cells

**Figure 2:**
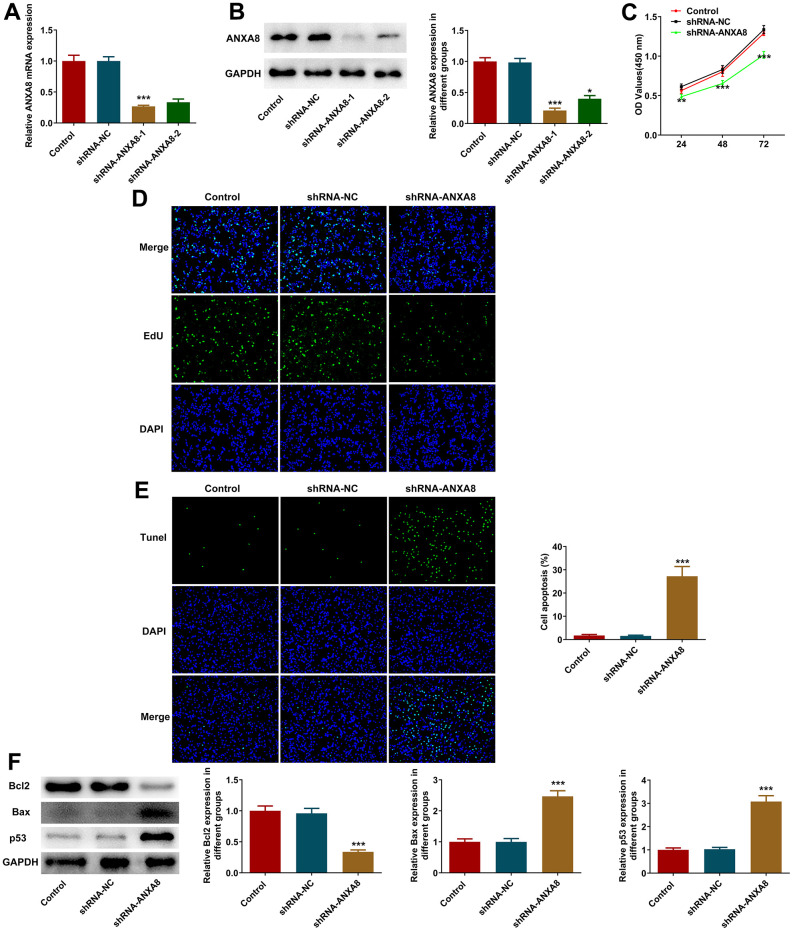
**Interference with ANXA8 inhibited the proliferation and promoted the apoptosis of CAOV3 cells.** (**A**) ANXA8 interference plasmid was constructed, and its interference efficacy was detected by RT-qPCR; (**B**) The transfection efficacy of ANXA8 interference plasmid was detected using western blotting; (**C**) CCK-8 assay was used to detect cell activity; (**D**) EdU staining was used to determine cell proliferation; (**E**) TUNEL staining was used to detect the level of apoptosis; (**F**) Western blot analysis of the expression of apoptosis-related proteins. *P<0.05, ***P<0.001 vs. shRNA-NC.

ANXA8 interference plasmid was constructed, and its interference efficacy was detected by RT-qPCR and western blotting (Figure 2A, 2B). The cells were divided into Control, shRNA-NC and shRNA-ANXA8 groups. CCK-8 was used to detect cell activity and EDU staining was used to detect cell proliferation. The results showed that compared with the shRNA-NC group, the cell activity and cell proliferation in the shRNA-ANXA8 group were significantly decreased (Figure 2C, 2D). TUNEL staining was used to detect the level of apoptosis, and the results showed that apoptosis was significantly increased in the shRNA-ANXA8 group compared with the shRNA-NC group (Figure 2E). Western blot analysis of the expression of apoptosis-related proteins showed that inhibiting the expression of ANXA8 in cells could significantly enhance the expression of pro-apoptotic proteins Bax and p53, and decrease the expression of Bcl-2 (Figure 2F).

### Interference with ANXA8 inhibited the invasion and migration of CAOV3 cells

**Figure 3:**
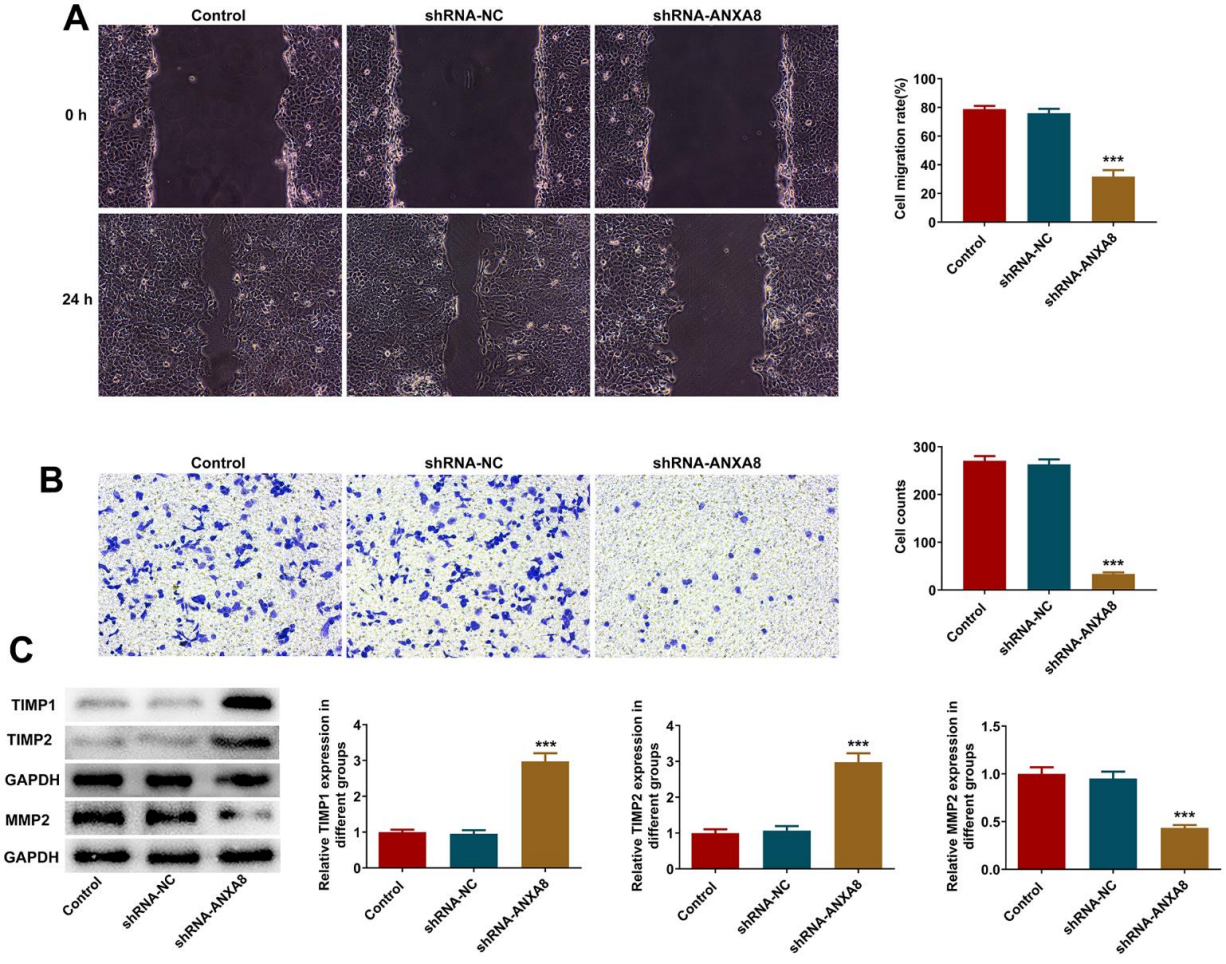
**Interference with ANXA8 inhibited the invasion and migration of CAOV3 cells.** (**A**) Wound healing assay was used to detect the migration ability of CAOV3 cells; (**B**) Transwell assay was used to detect the invasion ability of CAOV3 cells; (**C**) Western blot analysis of the expression of invasion and migration-related proteins TIMP1, TIMP2, and MMP2 in CAOV3 cells. ***P<0.001 vs. shRNA-NC.

Wound healing assay and transwell assay were used to detect invasion and migration abilities. We found that interference with the expression of ANXA8 in cells significantly decreased cell invasion and migration (Figure 3A, 3B). Western blot analyzed the expression of invasion and migration-related proteins TIMP1, TIMP2, and MMP2 and it was shown that compared with the shRNA-NC group, the expression of TIMP1 and TIMP2 in the shRNA-ANXA8 group was significantly increased and the expression of MMP2 was significantly decreased (Figure 3C).

### ANXA8 binds to UCHL5 in CAOV3 cells

**Figure 4:**
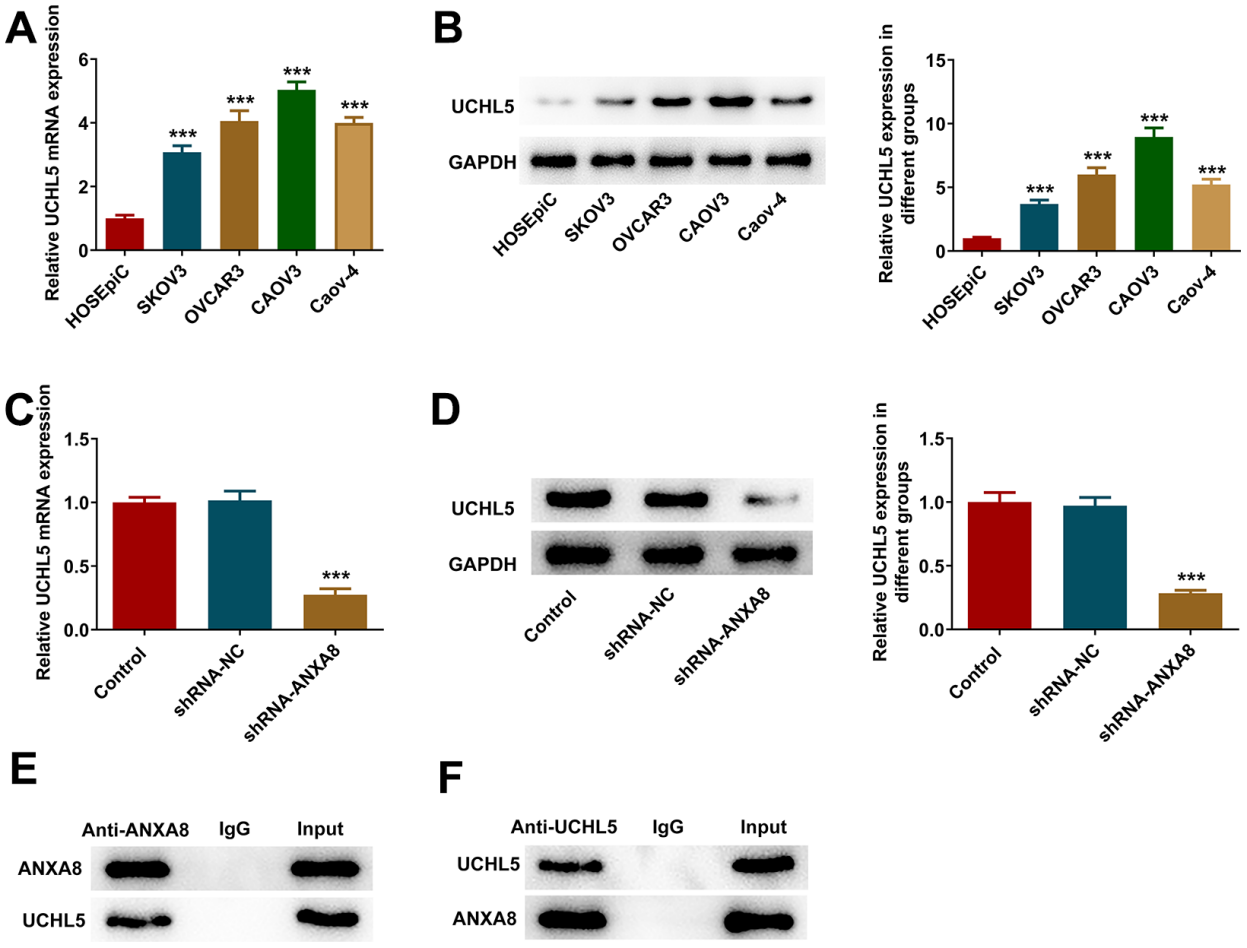
**ANXA8 bound to UCHL5 in CAOV3 cells.** (**A**) The expression of UCHL5 in the OC cell line was detected by RT-qPCR; (**B**) The expression of UCHL5 in the OC cell line was detected by western blotting. ***P<0.001 vs. HOSEpiC; (**C**) After interference with ANXA8, the expression of UCHL5 was detected by RT-qPCR; (**D**) After interference with ANXA8, the expression of UCHL5 was detected using western blotting; (**E**, **F**) The binding ability between ANXA8 and UCHL5 was verified by IP experiments. ***P<0.001 vs. shRNA-NC.

RT-qPCR and western blotting results suggested that UCHL5 expression was abnormally elevated in OC cell lines (Figure 4A, 4B). After interference with ANXA8, the expression of UCHL5 in cells was significantly decreased (Figure 4C, 4D). The binding ability between ANXA8 and UCHL5 was verified by IP experiments (Figure 4E, 4F).

### Interference with ANXA8 inhibited the proliferation, invasion and migration and promoted the apoptosis of CAOV3 cells through UCHL5

**Figure 5:**
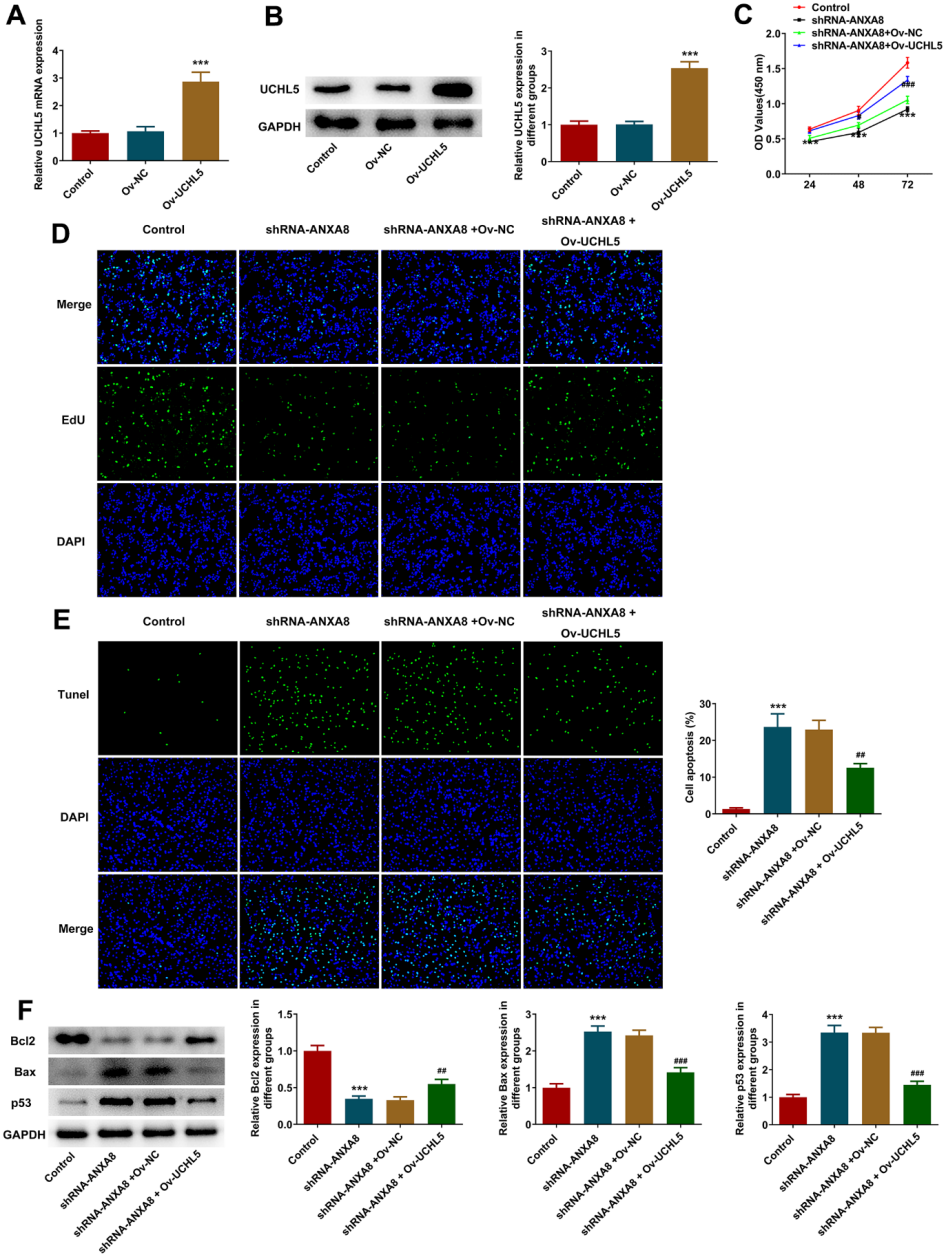
**Interference with ANXA8 inhibited the proliferation and promoted the apoptosis of CAOV3 cells through UCHL5.** (**A**) The overexpressed plasmid of UCHL5 was constructed, and its transfection efficiency was detected by RT-qPCR; (**B**) The overexpressed plasmid of UCHL5 was constructed, and its transfection efficiency was detected by western blotting; ***P<0.001 vs. Ov-NC. (**C**) CCK-8 was used to determine cell activity; (**D**) EDU staining was used to detect cell proliferation; (**E**) TUNEL staining was used to detect the level of apoptosis; (**F**) Western blot analysis of the expression of apoptosis-related proteins. ***P<0.001 vs. control; ##P<0.01, ###P<0.001 vs. shRNA-ANXA8+Ov-NC.

**Figure 6:**
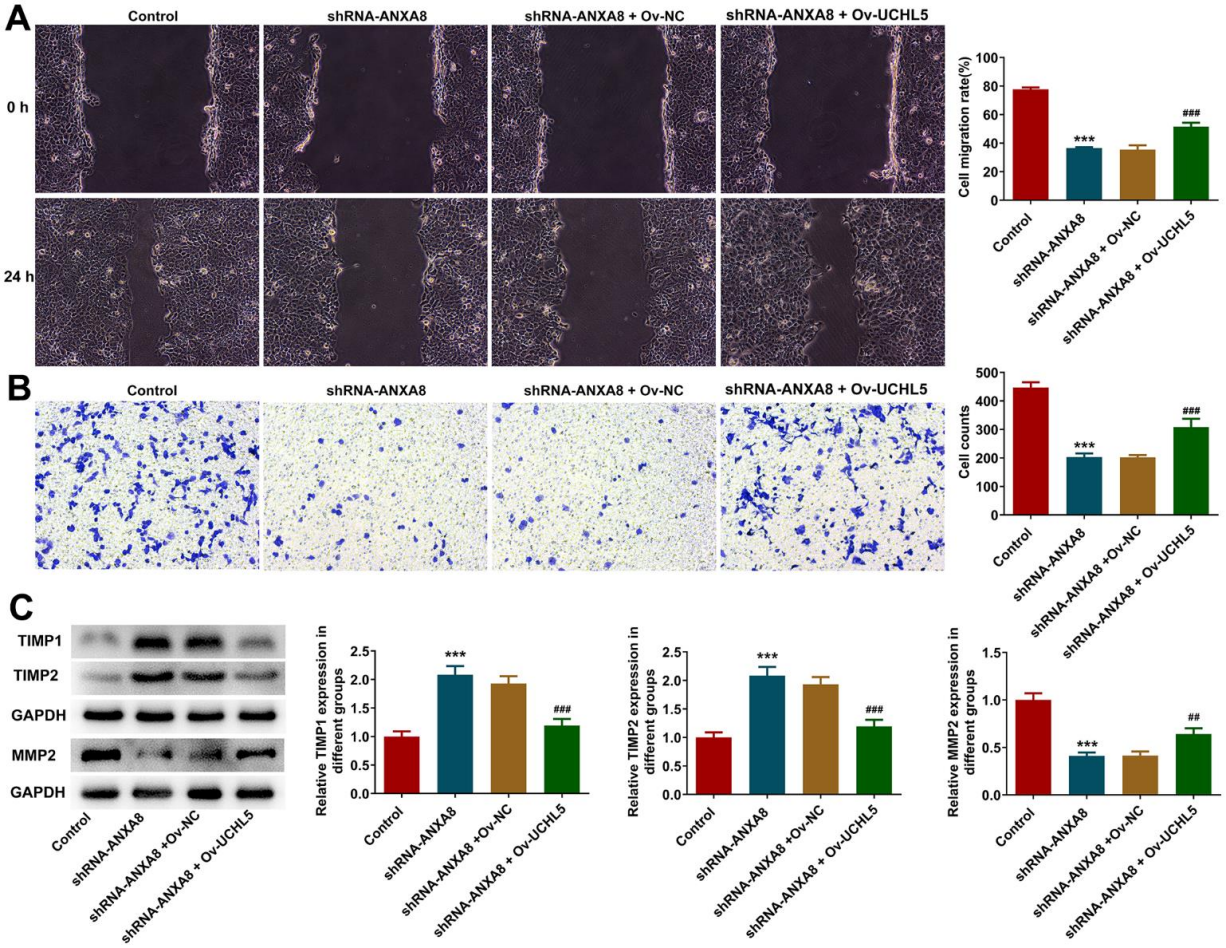
**Interference with ANXA8 inhibited the invasion and migration of CAOV3 cells through UCHL5.** (**A**) Wound healing assay was used to detect CAOV3 cell migration ability; (**B**) Transwell assay was used to detect CAOV3 cell invasion ability; (**C**) Western blot analysis of the expression of invasion and migration-related proteins TIMP1, TIMP2, and MMP2 in CAOV3 cells. ***P<0.001 vs. control; ##P<0.01, ###P<0.001 vs. shRNA-ANXA8+Ov-NC.

The overexpressed plasmid of UCHL5 was constructed, and then its transfection efficiency was evaluated using RT-qPCR and western blotting analysis (Figure 5A, 5B). The cells were divided into control, shRNA-ANXA8, shRNA-ANXA8+Ov-NC and shRNA-ANXA8 + Ov-UCHL5 groups. CCK-8 and EDU staining results showed that cell proliferation was significantly increased in the shRNA-ANXA8 + Ov-UCHL5 group compared with the shRNA-ANXA8+Ov-NC group (Figure 5C, 5D). The results of TUNEL staining and western blotting indicated that compared with the shRNA-ANXA8+Ov-NC group, apoptosis in the shRNA-ANXA8+Ov-UCHL5 group was significantly decreased (Figure 5E). This was accompanied by an increase in Bcl-2 protein and a decrease in the expression of Bax and p53 (Figure 5F). Wound healing assay, transwell assay and western blot results showed that the invasion and migration abilities of cells in the shRNA-ANXA8+Ov-UCHL5 group were significantly increased compared with those in the shRNA-ANXA8+Ov-NC group, accompanied with the down-regulated TIMP1, TIMP2 expression and the up-regulated MMP2 expression in the shRNA- ANXA8+Ov-UCHL5 group relative to the shRNA-ANXA8+Ov-NC group (Figure 6A–6C).

### Interference with ANXA8 inhibited the activation of Wnt/β-catenin signaling pathway via UCHL5

**Figure 7:**
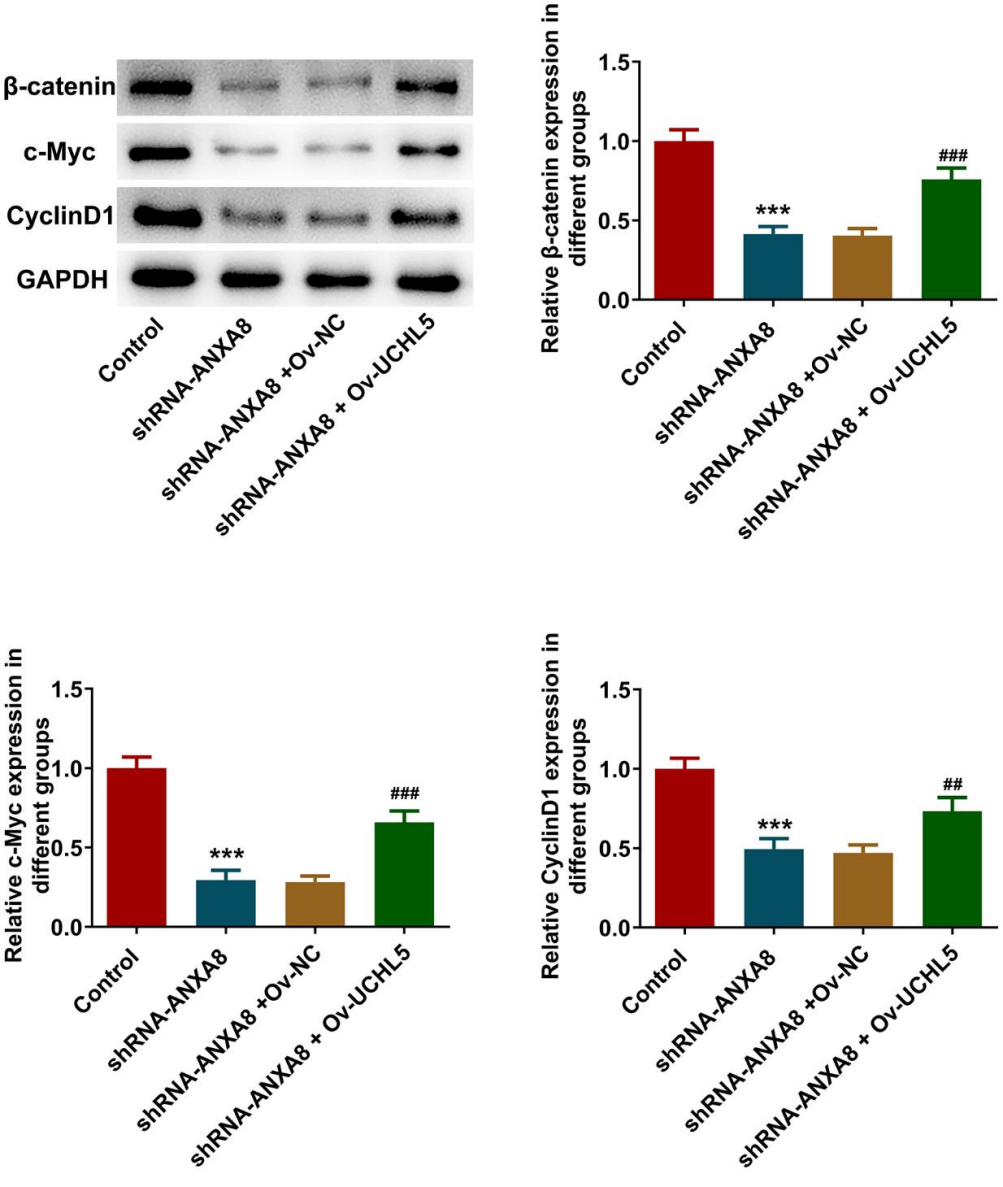
**Interference with ANXA8 inhibited the activation of Wnt/β-catenin signaling pathway via UCHL5.** Western blot analysis of Wnt/β-catenin signaling pathway-related proteins. ***P<0.001 vs. control; ##P<0.01, ###P<0.001 vs. shRNA-ANXA8+Ov-NC.

Western blot analysis revealed that inhibition of ANXA8 in cells significantly reduced the expression of β-catenin, c-Myc, and CyclinD1 proteins downstream of the Wnt/β-catenin signaling pathway, while further overexpression of UCHL5 reversed the expression of β-catenin, c-Myc, and CyclinD1 (Figure 7).

### Interference with ANXA8 inactivated the Wnt/β-catenin signaling pathway through UCHL5 to inhibit tumor growth in OC mice

**Figure 8:**
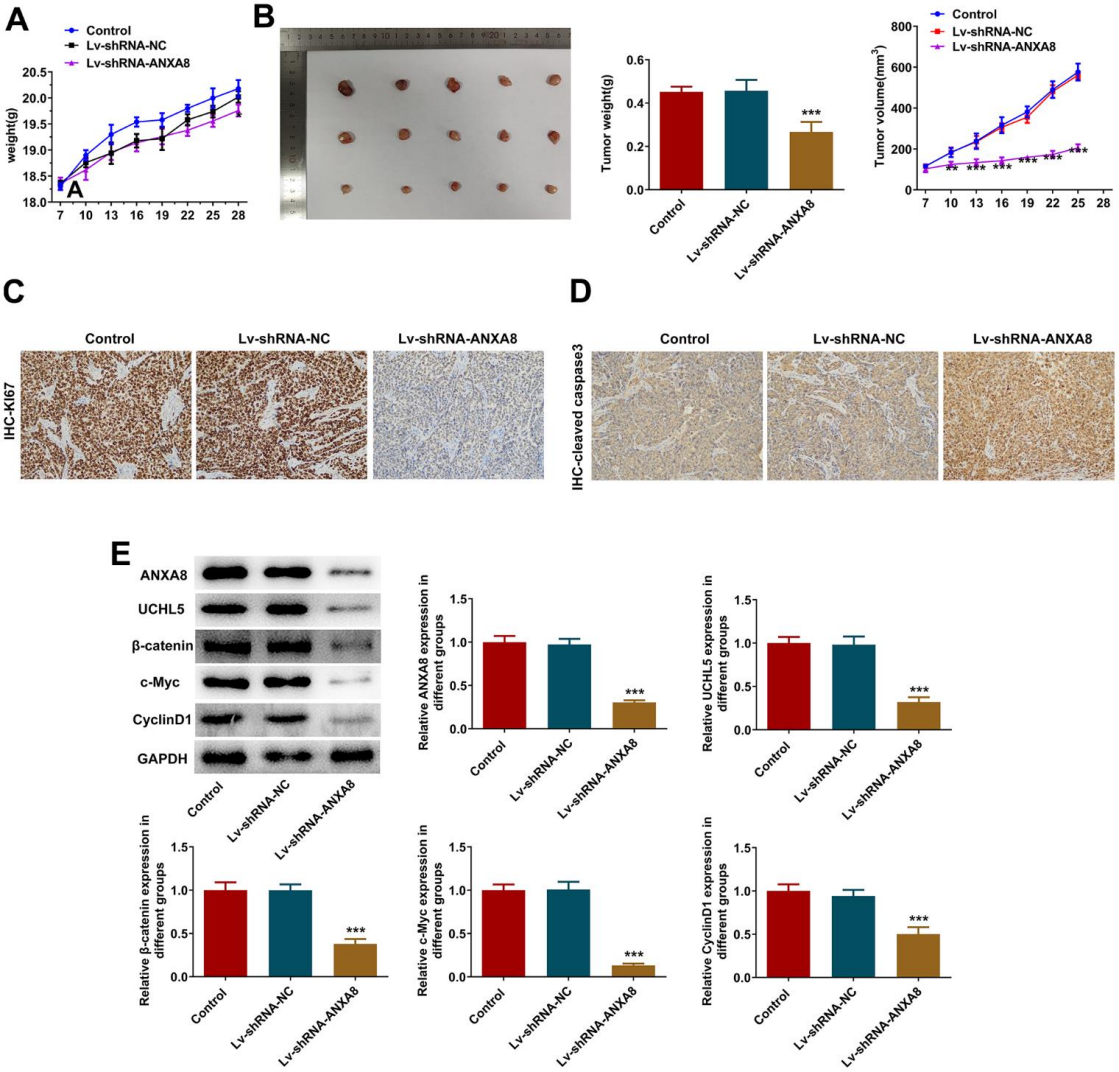
**Interference with ANXA8 inactivated the Wnt/β-catenin signaling pathway through UCHL5 to inhibit tumor growth in OC mice.** (**A**) The weight of the tumor in mice; (**B**) The volume of the tumor in mice; (**C**) IHC assay was used to detect the expression of KI67; (**D**) IHC assay was used to assess the expression of cleaved caspase3; (**E**) Western blotting was used to detect the expression of ANXA8, UCHL5, β-catenin, c-Myc, and CyclinD1 proteins.

The mice were divided into control group, Lv-shRNA-NC group and Lv-shRNA-ANXA8 group. The tumor weight and volume in mice are shown in Figure 8A, 8B. IHC detected KI67 and cleaved caspase3 expression and it was shown that inhibition of ANXA8 significantly inhibited the expression of KI67 in tumor tissues, and promoted the expression of cleaved caspase3 (Figure 8C, 8D). Western blot analysis of ANXA8, UCHL5, β-catenin, c-Myc, and CyclinD1 proteins showed that the expression of these proteins was inhibited after ANXA8 was interfered with (Figure 8E).

## DISCUSSION

OC ranks 7th in incidence and 8th in mortality among female cancers, posing a global challenge for modern medicine [13]. Complex intermolecular regulatory mechanisms are important pathological features of OC that urgently need to be further elucidated [14]. In this study, the activation of Wnt/β-catenin signaling pathway was further observed by interfering with ANXA8 and then inhibiting UCHL5 to observe the pathological manifestations of cell proliferation, invasion and migration of OC cells.

The abnormal expression of ANXA8 has a close association with the formation and progression of diverse neoplastic conditions, thereby serving as a predictive biomarker and a viable therapeutic target for biological interventions. [15–17]. Ma et al. have found through bioinformatics analysis that ANXA8 expression is abnormally elevated in lung cancer tissues, and ANXA8 overexpression is closely related to TNM stage and differentiation grade [18]. In gynaecological diseases, ANXA8 exhibits elevated mRNA expression in the porcine endometrium during days 11-13 of pregnancy, and ANXA8 stimulates the proliferation of porcine endometrial cells via the Akt signaling pathway [7]. High expression of ANXA8 promotes the progression of epithelial OC and predicts poor prognosis [19]. However, the specific regulatory mechanism of ANXA8 in OC has not been reported. In our experiments, we found that ANXA8 expression was abnormally elevated in OC cell lines. Inhibition of ANXA8 expression in OC cells could significantly inhibit cell proliferation, invasion, migration and promote apoptosis of cancer cells, which is consistent with a previous study [20]. This conclusion has also been verified in animal experiments. Therefore, targeting ANXA8 may be a novel treatment strategy for OC.

Biogrid database was used to predict the combination of ANXA8 and UCHL5. Our subsequent cell experiments also confirmed the association between ANXA8 and UCHL5. Ubiquitin carboxyl-terminal hydrolase L5 (UCHL5), as one of the isomers of carboxyl-terminal hydrolase of ubiquitin, participates in ubiquitination regulation of proteins and plays a crucial role in a variety of malignant tumors [21, 22]. A previous study showed that the higher the expression of UCHL5 in OC tissues, the worse the prognosis [23]. Our experiments also demonstrated that UCHL5 expression was abnormally elevated in OC cell lines. In addition, regulating TGF-β signaling by inhibiting UCHL5 expression and dephosphorylating Smad5 can inhibit the survival of TP2 mutant OC cells, thereby inducing cell apoptosis [24]. Furthermore, a study revealed that UCHL5 promotes cancer stemness and tumor progression in pancreatic adenocarcinoma (PAAD) by stabilizing ELK3, demonstrating its diversity in tumor regulation [25]. Therefore, we then explored whether ANXA8 could combine with UCHL5 to participate in the malignant process of OC cells. Moreover, overexpression of UCHL5 could significantly reverse the inhibited malignant progression in OC cells caused by interference with ANXA8. Therefore, the knockdown of ANXA8 inhibited the malignant progression of OC cells by inhibiting the expression of UCHL5.

The Wnt/β-catenin signaling pathway is closely related to cell proliferation and survival, and is known as a regulator of tumor proliferation and metastasis [26]. Furthermore, the Wnt/β-catenin signaling pathway has been shown to be involved in OC progression, and miR-27a activates Wnt/β-catenin signaling by targeting FOXO1, which promotes EMT in ovarian cancer [27]. Conversely, down-regulation of Wnt2B expression inhibits OC cell proliferation, invasion and angiogenesis by suppressing the Wnt/β-catenin signaling pathway [28]. It has been shown that inhibition of ANXA8 can inhibit the expression of Wnt/β-catenin [29]. In our experiments, we also found that inhibition of ANXA8 in cells led to a downregulation of proteins involved in the Wnt/β-catenin signaling pathway, indicating that the pathway was inhibited at this time. Meanwhile, it has been suggested that UCHL5 promotes the proliferation of endometrial cancer by activating Wnt/β-catenin signaling [30]. Overexpression of UCHL5 can significantly reverse the inhibition of ANXA8 on the Wnt/β-catenin signaling pathway. Thus, we came to a preliminary conclusion that interference with ANXA8 inhibited the activation of the Wnt/β-catenin signaling pathway through UCHL5, thereby inhibiting cell proliferation, invasion and migration of OC. In subsequent experiments, we will further elucidate the mechanism by adding inhibitors or activators of the pertinent signaling pathway.

## CONCLUSIONS

ANXA8 interference suppressed the activation of Wnt/β-catenin signaling pathway via UCHL5, leading to the inhibition of cell proliferation, invasion, and migration in OC, which might offer a new biomarker and a potential strategy for the management of OC.
